# Supplementary figures and images for: Cytokit: a single-cell analysis toolkit for high dimensional fluorescent microscopy imaging
Source: BMC Bioinformatics. 2019 Sep 2;20:448. doi: 10.1186/s12859-019-3055-3 (PMC6720861; doi:10.1186/s12859-019-3055-3)

## Before Quality Control Gating

## After Quality Control Gating

CD8

a

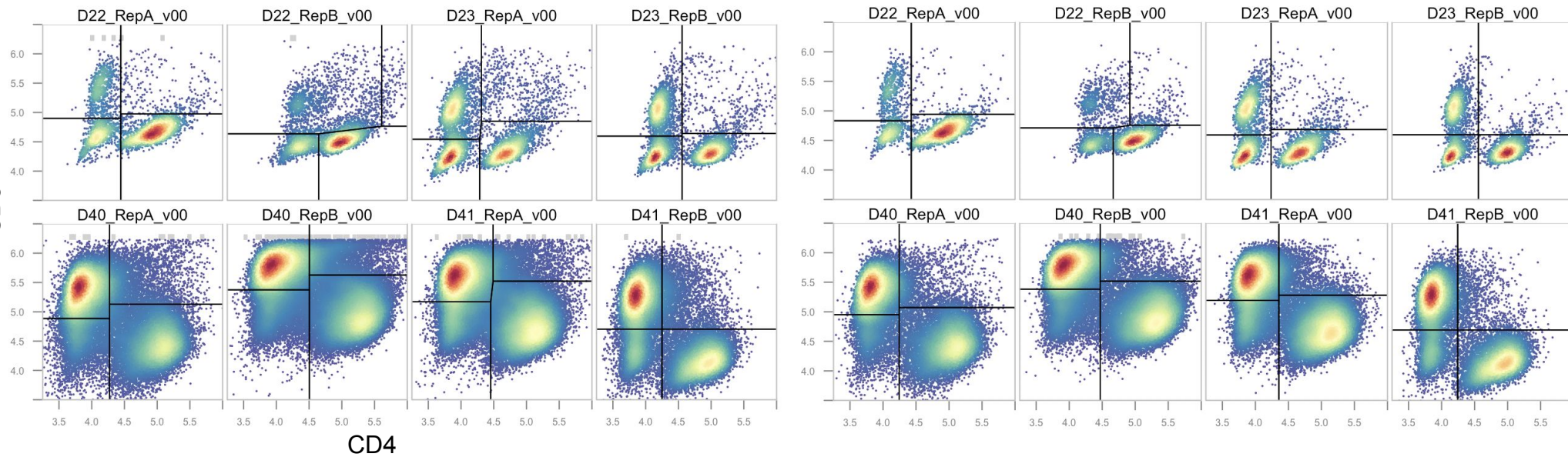

b

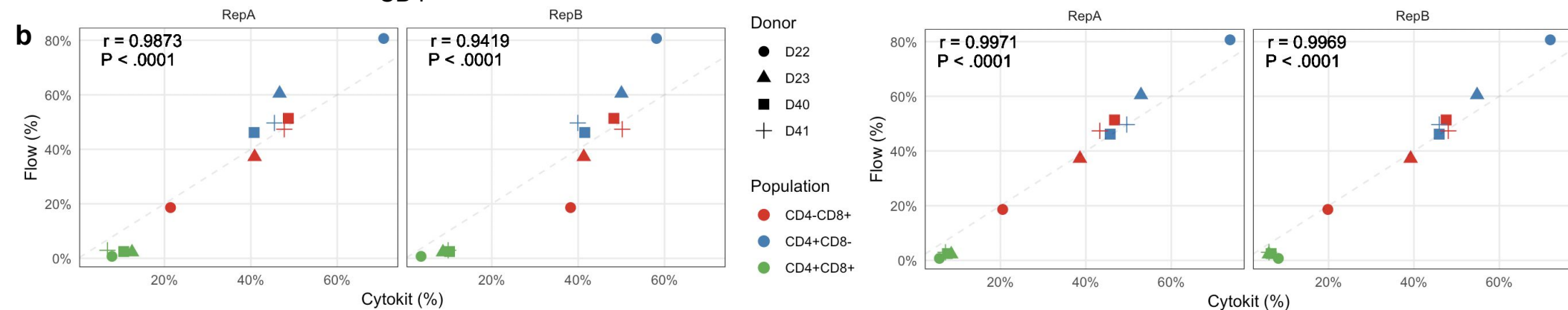

Supplement: Supplementary file 1 — Figure S1 Supplement to Fig. 8 demonstrating improvement in quantification after the inclusion of gates to remove debris and other segmentation artifacts. a CD4/CD8 cell populations for all 4 donors with both before and after groups including a terminal gate in the workflow to detect modes for each population, but all other gates left out in the “Before Quality Control Gating” example. b Cell population size for both replicates compared to flow cytometry measurements with improvement in correlation across populations after application of quality control gating (pearson correlation shown with significance from two-tailed t-test). (PDF 533 kb) [file 12859_2019_3055_MOESM1_ESM.pdf]

## Before Quality Control Gating

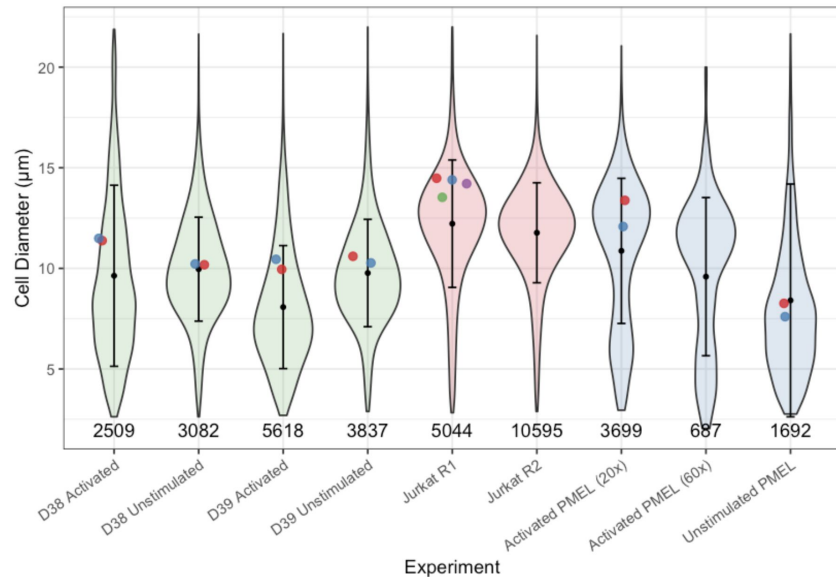

## After Quality Control Gating

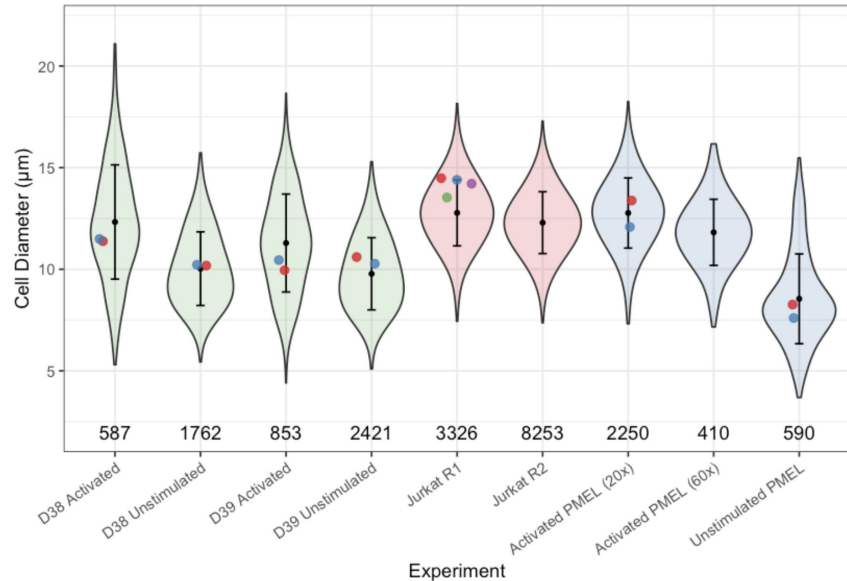

Supplement: Supplementary file 2 — Figure S2 Supplement to Fig. 10 demonstrating improvement in quantification after the inclusion of gates to remove debris and other segmentation artifacts. Cell counts from the two workflows are shown below each diameter distribution (violin with bars indicating +/− 1 s.d.) and corresponding Thermo Fisher cell counter diameter measurements are shown as colored dots. (PDF 371 kb) [file 12859_2019_3055_MOESM2_ESM.pdf]
